# Identification of CTA-Based Predictive Findings for Temporary and Permanent Neurological Dysfunction after Repair in Acute Type A Aortic Dissection

**DOI:** 10.1038/s41598-018-28152-z

**Published:** 2018-06-27

**Authors:** Hongliang Zhao, Didi Wen, Weixun Duan, Rui An, Jian Li, Minwen Zheng

**Affiliations:** 1 Department of Radiology, Xijing Hospital, Fourth Military Medical University, Xi’an, China; 2 Department of Cardiovascular Surgery, Xijing Hospital, Fourth Military Medical University, Xi’an, China

## Abstract

The aim of this study was to determine CT risk findings predictive of temporary neurological dysfunction (TND) and permanent neurological dysfunction (PND) after surgical repair for acute type A aortic dissection (ATAAD). A total of 255 patients (41 ± 16 years, 79% male) with ATAAD underwent aortic CT angiography (CTA) and surgical repair consecutively from January 2013 to June 2016. The CTA findings of the 255 patients for the thoracic aorta and carotid artery were analysed to identify risk factors predictive of TND and PND. Thirty-eight patients (15%) suffered TND, and 18 (7%) exhibited PND. Common carotid artery (CCA) dissection (OR = 4.63), lower enhancement of unilateral ICA (OR = 3.02) and aortic arch tears (OR = 2.83) were predictors of postoperative TND, while PND was best predicted by retrograde ascending aorta (aAO) dissection (OR = 5.62) and aortic arch tears (OR = 6.74). In CCA dissection, the extent of the entire CCA and proximal ICA (*P* = 0.014), a low-enhancement false lumen with re-entry (*P* = 0.000) and a severely narrowed true lumen without re-entry (*P* = 0.005) significantly increased the risk of postoperative TND. In patients with ATAAD, specific CT findings allow the individual risk of postoperative TND and PND to be identified and may guide subsequent surgical management.

## Introduction

Acute type A aortic dissection (ATAAD) is a life-threatening vascular emergency, mandating immediate surgical repair^1^. While developments in surgical, anaesthetic, and perfusion techniques have resulted in improved clinical outcomes, complex aortic arch procedures with hypothermic circulatory arrest (HCA), selective cerebral perfusion (SCP) and individual arterial cannulation modalities are still associated with a relatively high incidence of postoperative neurological complications and mortality^2–4^.

Temporary neurological dysfunction (TND) and permanent neurological dysfunction (PND) represent the major neurological complications associated with surgical repair. Several previous studies^3,5–7^ have been undertaken to examine preoperative and procedural predictors of TND and PND as well as mortality after operations to address ATAAD. In addition to procedure-related risk factors, negative neurological outcomes are often related to the preoperative condition of cerebral malperfusion of the patient^7–10^. However, adequate risk stratification of ATAAD patients based on the preoperative algorithm, especially in an emergency situation, is difficult due to the absence of apparent symptoms and signs for the surgeon to evaluate^11^. Thus, a number of modalities need to be applied to monitor the current status of patients with ATAAD to enable individual treatment and meticulous attention to critical procedural details.

According to the guidelines for addressing such conditions^12^, CT is the most commonly used imaging technique for the evaluation of acute aortic syndromes and for aortic dissection in particular because of its speed, widespread availability, and excellent sensitivity of >95% for aortic dissection. Early imaging using CT may very well be contributing to the accurate detection of cerebral malperfusion based on associated CT angiography (CTA) findings for the aorta and its branches^13^. Although different risk factors for postoperative TND and PND as well as mortality have been evaluated in patients with ATAAD^5–9^, the correlation between CTA findings in ATAAD and neurological complications has rarely been discussed.

Here, we hypothesize that specific CTA findings in ATAAD are associated with postoperative neurological dysfunction. If so, CTA imaging may help individualize perioperative management and focus meticulous attention on critical procedural details. Thus, the aim of the present study was to determine the predictive value of CTA risk findings for postoperative TND and PND.

## Results

### Patient Characteristics

The demographics, clinical features, surgical procedures and intraoperative data of the subjects in the three groups are displayed in Table 1. The only significant difference between the groups was the occurrence of acute renal failure. There were no significant differences in terms of age, sex, body mass index, previous medical history, surgical procedures, cerebral protection method or operation time between the three groups (*P* > 0.05 for all comparisons).

**Table 1:** Baseline Characteristics.

Characteristics	NND group (*n* = 199)	TND group (*n* = 38)	PND group (*n* = 18)
Age	47.5 ± 10.3	49.1 ± 9.4	50.0 ± 6.9
Male	156 (78.4)	30 (78.9)	15 (83.3)
Body mass index (kg/m^2^)*	24.4 ± 3.1	23.7 ± 3.4	23.5 ± 9.2
Hypertension	146 (73.4)	28 (73.7)	16 (88.9)
Smoking	88 (44.2)	18 (47.5)	8 (44.4)
Marfan’s syndrome	3 (1.5)	0 (0)	0 (0)
Diabetes mellitus	5 (2.5)	2 (5.3)	0 (0)
Dyslipidaemia	92 (45.2)	16 (42.1)	8 (44.4)
Coronary artery disease	11 (5.5)	2 (5.3)	1 (5.6)
Chronic obstructive lung disease	2 (0.8)	0 (0)	1 (5.6)
Cerebrovascular disease	44 (22.1)	10 (26.3)	4 (22.2)
Previous operation on the aorta	8 (4.0)	2 (5.3)	1 (5.6)
Chronic cerebral infarction	27 (13.6)	6 (15.8)	3 (16.7)
Preoperative characteristics			
Acute cerebral infarction (MR + )	19 (19.9)	10 (38.5)	4 (36.4)
Left ventricular ejection fraction < 40%	18 (0.3)	1 (2.6)	0 (0)
Acute renal failure	22 (11.1)	10 (26.3)	7 (38.9)
Surgical Procedures			
Bentall procedure^†^	153 (76.9)	30 (78.9)	16 (88.9)
Cabrol procedure^‡^	11 (5.5)	1 (2.6)	0 (0)
Ascending aorta replacement	35 (17.6)	7 (18.4)	2 (11.1)
Total arch replacement	187 (94.0)	37 (97.4)	18 (100)
Hemiarch replacement	12 (6.0)	1 (2.6)	0 (0)
Stented elephant trunk	187 (94.0)	37 (97.4)	18 (100)
Coronary artery bypass graft	6 (3.0)	1 (2.6)	1 (5.6)
Unilateral SACP	199 (100)	38 (100)	18 (100)
Operation times*			
CPB time (min)	210.1 ± 41.9	232.9 ± 46.3	220.1 ± 31.6
Aortic cross-clamp time (min)	98.8 ± 22.4	102.0 ± 21.5	107.5 ± 32.7
HCA time, (min)	30.2 ± 8.3	32.5 ± 7.4	31.3 ± 6.5

Note.-Except where indicated, the data are numbers of patients, with percentages in parentheses.

*The data are the means ± standard deviations.

NND = no neurological dysfunction; TND = temporary neurological dysfunction; PND = permanent neurological dysfunction; SACP = selective antegrade cerebral perfusion; CPB = cardiopulmonary bypass; HCA = hypothermic circulatory arrest.

^†^A Bentall procedure is a cardiac operation involving composite graft replacement of the aortic valve, aortic root and ascending aorta, with re-implantation of the coronary arteries into the graft. The Bentall procedure was first described in 1968 by Hugh Bentall and Antony De Bono.

^‡^A Cabrol technique is a cardiac operation in which the coronary ostia are anastomosed to a second graft in an end-to-end fashion, and this graft is then attached to the ascending aortic conduit side-to-side.

### Neurological Complications

The overall incidence of postoperative neurological complications after aortic repair was 22% (56/255). In-hospital postoperative PND occurred in 18 out of 255 patients (7.1%), and TND was detected in 38 patients (14.9%). Four patients with PND (22.2%, 4/18) died, but none of the patients with TND died.

### CTA Findings for Analysis

Detailed CTA findings that were considered to represent possible risk factors for postoperative TND and PND are presented in Tables 2 and 3. The CTA findings included in the analysis are as follows: diameter of the involved aAO, retrograde dissection in the aAO, entry tears in the aAO, diameter of aAO tears, aortic sinus dissection, entry tears in the aortic arch, diameter of aortic arch tears, entry tears in the descending aorta, common carotid artery (CCA) dissection, lower enhancement in the unilateral ICA, origin of CCA in the false lumen, VA dissection, and lower enhancement of unilateral VA.

**Table 2:** Univariate Analysis of CTA Risk Findings for Postoperative TND.

CTA Findings	Univariate Analysis			Multivariate Analysis		
	NND group (*n* = 199)	TND group (*n* = 38)	P Value	Odds Ratio	95% CI	*P* Value
Diameter of the involved aAO (mm)*	51.2 ± 10.6	50.5 ± 8.0	0.700			
Retrograde dissection in the aAO	27 (19.6)	6 (28.9)	0.717			
Entry tear in the aAO	172 (86.4)	32 (84.2)	0.717			
The diameter of aAO tear (mm)*	1.96 ± 1.6	2.0 ± 1.0	0.948			
Aortic sinus dissection	63 (31.7)	12 (31.6)	0.992			
Entry tear in the aortic arch	100 (50.3)	29 (76.3)	0.003	2.827	1.084–7.368	0.034
Diameter of the aortic arch tear (mm)*	11.6 ± 8.5	8.9 ± 8.1	0.133			
Entry tear in the descending aorta	39 (19.6)	2 (5.3)	0.032	0.306	0.056–1.661	0.170
CCA dissection	54 (27.1)	24 (63.2)	0.000	4.626	2.094–10.219	0.000
CCA originating from false lumen	2 (1.01)	2 (5.3)	0.062			
Lower enhancement in unilateral ICA	21 (10.6)	12 (31.6)	0.001	3.019	1.182–7.707	0.021
VA dissection	13 (6.5)	2 (5.3)	0.768			
Lower enhancement in unilateral VA	17 (8.5)	3 (7.9)	0.895			

Note.-Except where indicated, the data are numbers of patients, with percentages in parentheses.

*The data are the means ± standard deviations.

NND = no neurological dysfunction; TND = temporary neurological dysfunction; aAO: ascending aorta; CCA = common carotid artery; ICA = internal carotid artery; VA = vertebral artery.

**Table 3:** Analysis of CTA Risk Findings for Postoperative PND.

CTA Findings	Univariate Analysis			Multivariate Analysis		
	NND group (*n* = 199)	PND group (*n* = 18)	*P* Value	Odds Ratio	95% CI	*P* Value
The diameter of involved aAO (mm)*	51.2 ± 10.6	53.1 ± 11.0	0.471	5.622	1.788–17.679	0.003
Retrograde dissection in the aAO	27 (19.6)	8 (44.4)	0.002			
Entry tear in the aAO	172 (86.4)	10 (55.6)	0.002			
The diameter of aAO tear (mm)*	1.96 ± 1.6	1.6 ± 0.9	0.500			
Aortic sinus dissection	63 (31.7)	8 (44.4)	0.268			
Entry tear in the aortic arch	100 (50.3)	15 (55.6)	0.007	6.742	1.736–26.184	0.006
The diameter of aortic arch tear (mm)*	11.6 ± 8.5	11.1 ± 6.9	0.816			
Entry tear in the descending aorta	39 (19.6)	4 (22.2)	0.789			
CCA dissection	54 (27.1)	8 (44.4)	0.120			
Lower enhancement in unilateral ICA	21 (10.6)	16 (88.9)	0.031	3.340	0.887–12.570	0.075
CCA originating from false lumen	2 (1.01)	0 (0)	0.669			
VA dissection	13 (6.5)	1 (5.6)	0.872			
Lower enhancement in unilateral VA	17 (8.5)	1 (5.6)	0.660			

Note.-Except where indicated, data are numbers of patients, with percentages in parentheses.

*The data are the means ± standard deviations.

NND = no neurological dysfunction; PND = permanent neurological dysfunction; aAO = ascending aorta; CCA = common carotid artery; ICA = internal carotid artery; VA = vertebral artery.

The number and percentage of each CTA finding between the TND, PND and no neurological dysfunction (NND) groups are also presented in Tables 2 and 3. The diameters of the involved aAO (51.2 mm ± 10.6 vs. 50.5 mm ± 8.0 or 53.1 mm ± 11.0, both *P* > 0.05), aAO tears (17.2 mm ± 7.4 vs. 20.5 mm ± 11.1 or 15.8 mm ± 10.0, both *P* > 0.05), and aortic arch tears (11.6 mm ± 8.5 vs. 8.9 mm ± 8.1 or 11.1 mm ± 6.9, both *P* > 0.05) were similar in the NND, TND and PND groups. A significantly higher incidence of entry tears in the aortic arch was found in both the TND (76.3%) and PND groups (83.3%) than in the NND group (50.3%, *P* = 0.003 and *P* = 0.007). In addition, lower enhancement in the unilateral ICA was significantly increased in both the TND (31.6%) and PND groups (88.9%) compared with that in the NND group (10.6%, *P* = 0.001 and *P* = 0.000). Furthermore, significantly more patients exhibited CCA dissection in the TND group (63.2%) than in the NND group (27.1%, *P* = 0.000), whereas the number of entry tears in the dAO was significantly lower in patients with TND (5.3%) than in the NND subjects (19.6%, *P* = 0.03). A significantly higher incidence of retrograde dissection in the aAO was also found in the PND group (44.4%) than in the NND cohort (13.6%, *P* = 0.002).

### CTA Risk Findings for TND

**Figure 1:**
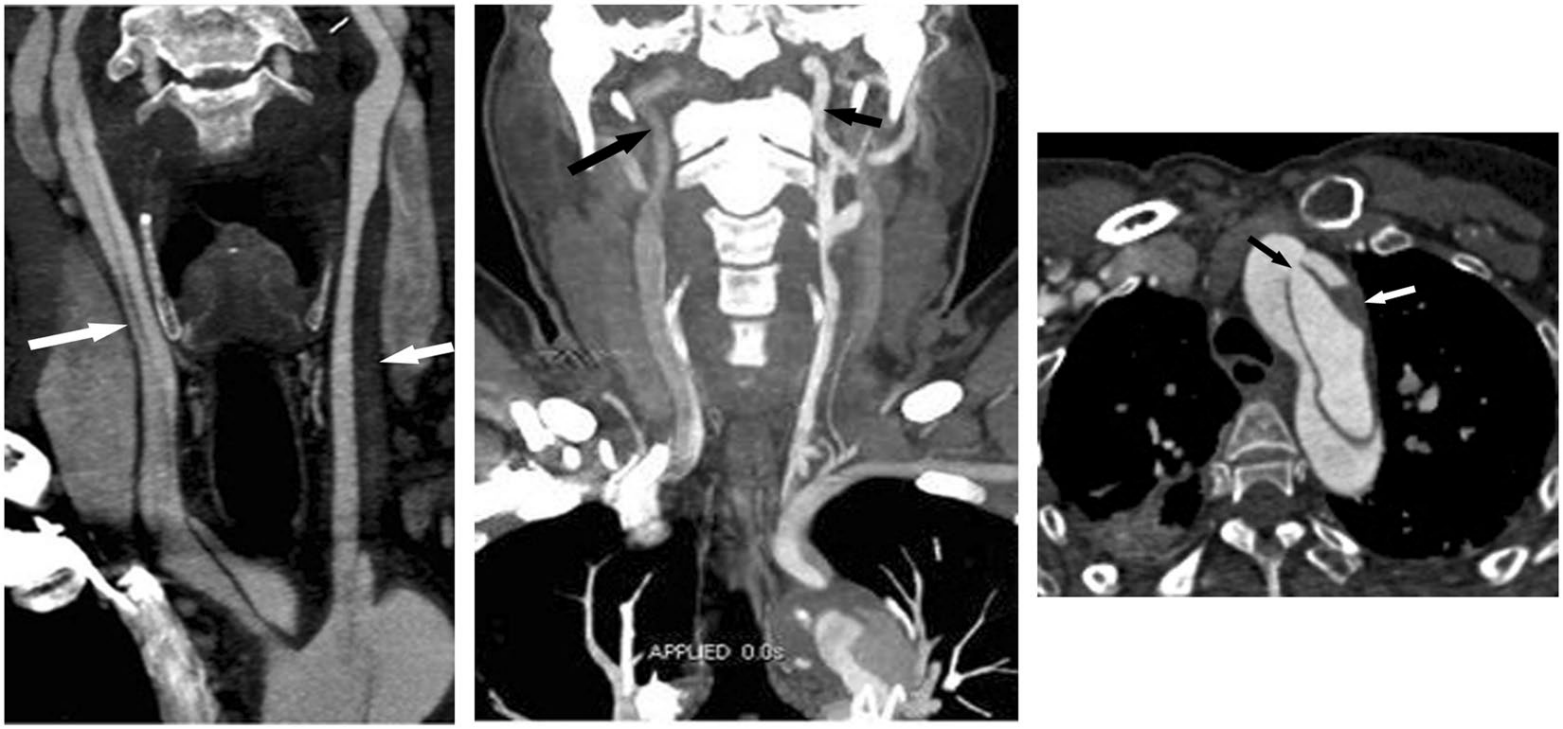
Three CTA Risk Findings for Postoperative TND. (a) Carotid and aortic CTA in a 39-year-old man with ATAAD. Coronal thin MIP image showing aortic dissection extending to both the right (long arrow) and left CCA (short arrow). This finding suggests that dissection involving CCA is an important risk predictor for postoperative TND. (b) Carotid and aortic CTA in a 54-year-old man with ATAAD. Coronal thin MIP image showing that the contrast enhancement of the right ICA (long arrow) is lower than that of the left ICA (short arrow), due to the origin from the false lumen of the right CCA dissection. The results show an inadequate blood supply in the unilateral carotid artery and imply ipsilateral cerebral hypoperfusion. (c) Carotid and aortic CTA in a 49-year-old man with ATAAD. Axial image showing an entry tear located in the aortic arch (black arrow). Note the low density of thrombosis (white arrow) in the false lumen. An aortic arch tear implies an increased risk of pre- and intraoperative embolism into the cerebral artery via the entry point. CTA = CT angiography; TND = temporary neurological dysfunction; ATAAD = Acute type A aortic dissection; MIP = maximum intensity projection; ICA = internal carotid artery; MPR = multiplanar reformation. Note the low-density haematoma in the false lumen of the involved left CCA.

Univariate analysis of CTA risk findings revealed that an entry tear in the aortic arch or the descending aorta, CCA dissection, and lower enhancement in the unilateral ICA were implicated in the occurrence of postoperative TND, as shown in Table 2. The following multivariate analysis, including significant preoperative acute renal failure, revealed that CCA dissection (OR = 4.63, *P* = 0.000), lower enhancement in the unilateral ICA (OR = 3.02, *P* = 0.021), and an entry tear in the aortic arch (OR = 2.83, *P* = 0.034) were independent CTA risk predictors for postoperative TND (Table 2, Fig. 1).

### CTA Risk Findings for PND

**Figure 2:**
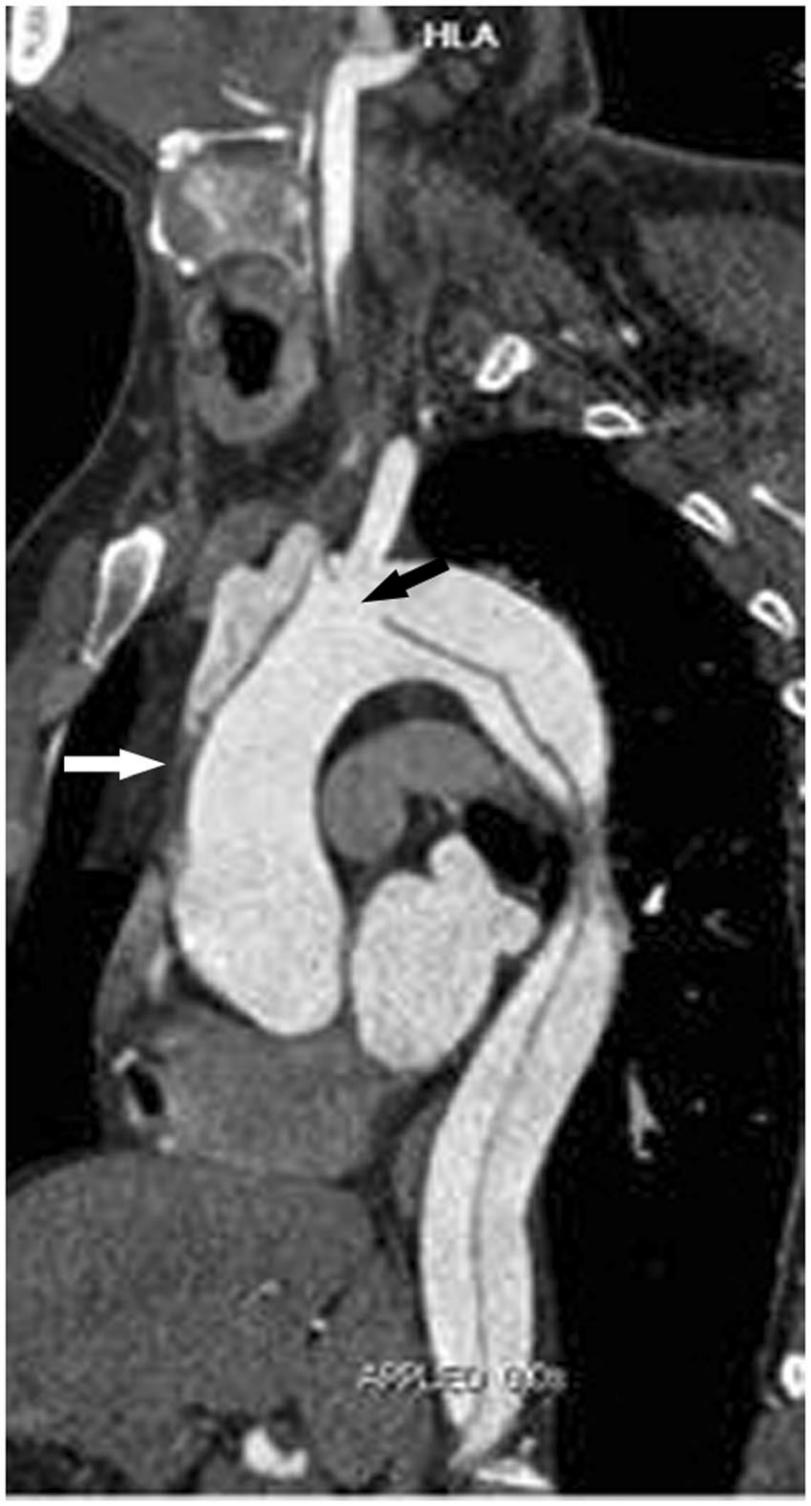
Two CTA Risk Findings for Postoperative PND. Oblique sagittal MPR image showing dissection involving the thoracic aorta and supra-aortic branches. An entry tear (black arrow) is located in the aortic arch, and the intimal flap extends into the aAO in a retrograde fashion. Note the thrombosis (white arrow) in the false lumen, due to the integrated intimal flap in the aAO. The two CTA findings, which exist simultaneously, indicate an extremely high risk of thrombus in the false lumen extending to the cerebrum via the entry point. CTA = CT angiography; PND = permanent neurological dysfunction; MPR = multiplanar reformation; aAO = ascending aorta.

Univariate analysis of CTA risk findings revealed that retrograde dissection of the aAO, an entry tear in the aAO or aortic arch, and low attenuation in the unilateral ICA are implicated in the occurrence of postoperative PND, as shown in Table 3. After multivariate analysis, the significant independent CTA risk predictors of postoperative PND were retrograde dissection in the aAO (OR = 5.62, *P* = 0.003) and an entry tear in the aortic arch (OR = 6.74, *P* = 0.006) (Table 3, Fig. 2).

### Correlation between CTA Characteristics of CCA Dissection and TND

**Figure 3:**
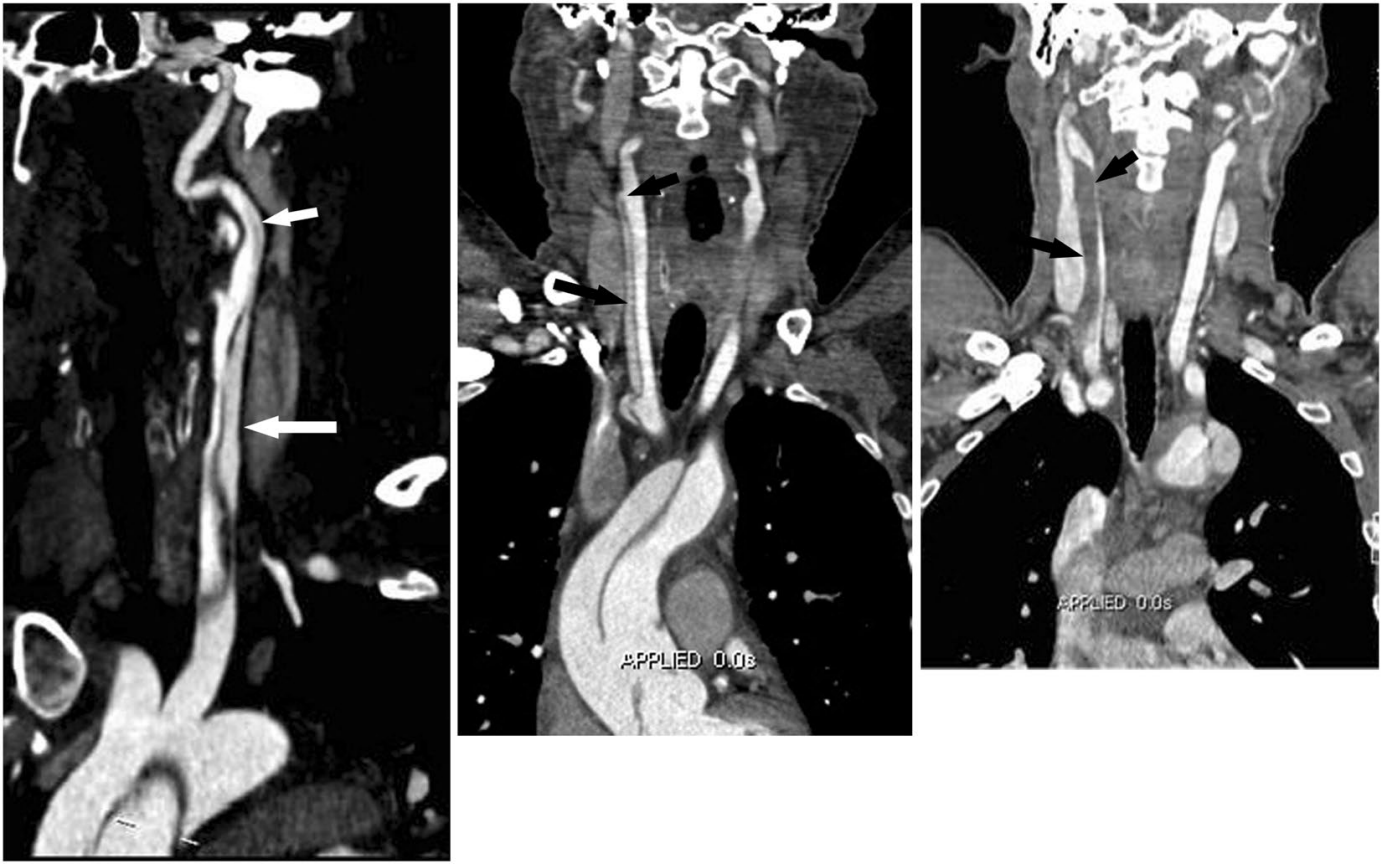
Significant Detailed Findings of CCA Dissection for Postoperative TND. (a) CPR image showing aortic dissection involving the left CCA (long arrow) and proximal ICA (short arrow). Patients with a CCA dissection longer in length involving the entire CCA and proximal ICA exhibit a significantly higher incidence of postoperative TND than those with CCA dissection involving either the CCA alone or the proximal CCA. (b) Coronal MPR image showing aortic dissection involving the right CCA (long arrow) with a re-entry tear (short arrow) in the distal CCA. Note that the density of the false lumen is lower than that of the true lumen. This finding indicates a higher risk of the thrombus in the low-enhancement false lumen extending to the cerebrum via the re-entry point. (**c**) Coronal MPR image showing aortic dissection extending into the right CCA. CCA dissection without re-entry in the distal CCA and thrombosis in the false lumen (arrow). The severely narrowed true lumen (arrow head) resulting from the compression of a haematoma in the false lumen implies probable cerebral hypoperfusion due to a decreased blood supply from the true lumen of the CCA. CTA = CT angiography; CPR = curved multiplanar reformation; CCA = common carotid artery; MPR = multiplanar reformation.

Because CCA dissection was indicated to be an independent CTA risk finding for postoperative TND, the detailed CTA characteristics of the involved CCA were further analysed to identify the correlation between these findings and TND. The location, extension and characteristics of the CCA dissections are shown in Table 4. There was no significant difference in the location of the dissection on the right side, left side or bilateral CCA between the TND and NND groups (all *P* > 0.05). The extension of dissections involving the proximal CCA and the entire CCA did not differ significantly between the two groups, whereas patients in the TND group exhibited a higher incidence of dissections involving the entire CCA and proximal ICA (*P* = 0.014) (Fig. 3). When the dissection involved the CCA, re-entry in the CCA combined with visually lower enhancement or low attenuation matter in the false lumen (Fig. 3), or no re-entry in the CCA combined with a severely narrowed true lumen compressed by a thrombosed false lumen (Fig. 3) increased the risk of postoperative TND.

**Table 4:** Detailed CTA Characteristics of the Involved CCA between NND and TND Groups

CTA Characteristics	NND group (*n* = 58)	TND group (*n* = 26)	*P* Value
The location of CCA dissection			
Bilateral	14 (24.1)	6 (23.1)	0.556
Unilateral	44 (75.9)	20 (76.9)	0.556
Left	9 (15.5)	3 (11.5)	0.630
Right	35 (60.3)	17 (65.4)	0.660
Extension of CCA dissection			
Proximal CCA	14 (24.1)	5 (19.2)	0.619
Whole CCA	41 (70.7)	15 (57.7)	0.243
Whole CCA + proximal ICA	3 (5.2)	6 (23.1)	0.014
Detailed findings of CCA dissection			
With re-entry			
Same enhancement in the true and false lumen	29 (50.0)	5 (19.2)	0.008
Lower enhancement in the false lumen, n (%)	2 (3.4)	11 (42.3)	0.000
No re-entry			
Without severe narrow in the true lumen	23 (39.7)	2 (7.7)	0.003
Severely narrowed true lumen by thrombosed false lumen	4 (6.9)	8 (30.8)	0.005

Note.-The data are numbers of patients, with percentages in parentheses.

NND = no neurological dysfunction; TND = temporary neurological dysfunction; CCA = common carotid artery; ICA = internal carotid artery.

## Discussion

In this retrospective study of patients with ATAAD, we observed that CCA dissection, lower enhancement in the unilateral ICA, and aortic arch tears are independent CTA risk predictors for postoperative TND, while both retrograde aAO dissection and aortic arch tears are independent CTA risk predictors for PND. The extent of the entire CCA and proximal ICA involved, CCA re-entry and lower enhancement in the false lumen, and a severely narrowed true lumen in the CCA without re-entry significantly increased the risk of TND when the CCA was involved.

Postoperative neurological complications, including TND (with a reported incidence of 2.5–33%) and PND (with an incidence of 4.2–24%), remain challenging for patients with ATAAD^3,5,7,14–16^. In our study, the incidences of TND and PND were 14.9% and 7.1%, respectively, which are lower than those reported in most other studies. This discrepancy likely occurred because all operations were performed at our high-volume cardiovascular surgical centre with dedicated high-volume thoracic aortic surgeons. Moreover, all patients in our centre are cared for via an emergent green route, and the majority undergo emergency surgical repair within 8 hours of admission.

To increase sensitivity in the identification of risk factors, previous authors^3,5–7^ have elected to combine PND and death as adverse outcomes (AO) for evaluation, but this approach may have confused the real risk predictors for PND or mortality. The most common causes of postoperative mortality involve cardiac aetiology, while neurological complications are less significant^17^. Therefore, only PND was evaluated in the present study.

As we suspected, CCA dissection was shown to be a major CTA risk predictor for TND but not for PND. A previous study^13^ indicated that carotid artery dissection may well correlate with cerebral malperfusion, which presents strong potential for adverse outcomes in postoperative patients with ATAAD. In most cases requiring extensive arch repair, such as total arch replacement, unilateral antegrade selective cerebral perfusion is the cerebral protection method of choice^18^. In the setting of right CCA dissection with severe stenosis of the true lumen, however, bilateral two-vessel perfusion through the right axillary artery or the innominate artery and the left common carotid artery may be essential to avoid left cerebral artery insufficiency, which may result in cerebral infarction.

The detailed CTA characteristics of CCA dissection need to be carefully considered. Lower enhancement in the false lumen of the CCA with re-entry or a severely narrowed true lumen of the CCA without re-entry is significantly more common in patients with TND. A longer extent of dissection of the CCA and proximal ICA also increase the risk of TND compared with dissection of the proximal CCA or entire CCA.

Lower enhancement in the unilateral ICA is potentially predictive of TND. Several conditions contribute to this finding, such as the ICA originating from the false lumen, a severely narrowed true lumen of the CCA, or low-enhancement CCA originating from the false lumen of the IA. Lower enhancement indicates decreased blood flow in the carotid artery, which may be highly predictive of cerebral hypoperfusion. This finding suggests a therapeutic strategy that may resolve malperfusion^13,19^. Lower enhancement in the unilateral ICA also shows a high incidence of 88.9% in patients with PND. Therefore, the finding that this condition is not an independent predictor of PND was unexpected. This unexpected result may have occurred because this condition, which represents a manifestation of ipsilateral cerebral hypoperfusion, can be resolved via rapid establishment of cerebral flow and surgical repair of the dissection.

In our study, an aortic arch tear was found to significantly increase the risk of both TND and PND. It is reasonable to presume that emboli (thrombi in the false lumen or plaques ruptured during a surgical procedure) flow more easily into craniocervical arteries via the entry point, resulting in subsequent artery occlusion and cerebral infarction. Recent studies have demonstrated that strokes in patients with cervical artery dissection are most frequently associated with artery-to-artery embolization^20,21^. In the case of an aortic arch tear combined with a thrombus in the false lumen, CTA information could alert the surgeon to pay meticulous attention to critical procedural details to avoid dislodging the embolus from the tear. The incidence of stroke is likely to be effectively reduced by following these guidelines. Although dAO tears were excluded as a risk predictor due to their low occurrence, we still believe such tears have an effect on postoperative neurological dysfunction due to sharing similar mechanisms with aortic arch tears.

The finding that retrograde aAO dissection serves as an independent predictor of PND was unexpected. This condition is commonly associated with an aortic arch or dAO tear. Thrombogenesis in the false lumen is common due to the integrated intimal flap. Therefore, avoiding the dislodgment of a thrombus during surgical procedures may effectively reduce postoperative PND.

This study was limited by its retrospective nature. Additionally, there was some selection bias as well as a sample size and character mismatches between the three groups. The sample size of the patient groups with postoperative TND and PND was also small, which may influence the accuracy of the outcomes. Furthermore, our study focused on only in-hospital TND and PND, and long-term clinical outcomes may be more important for elucidating real CTA risk predictors. Finally, our CTA scanning procedure did not include the head, which might provide more useful information regarding the status of cerebral malperfusion.

The findings of the present study indicated that specific carotid and aortic CTA findings may help predict individual risk for postoperative TND and PND in patients with ATAAD, in addition to providing guidance for individual surgical strategies that may decrease postoperative neurological dysfunction.

## Methods

### Patient Characteristics

The institutional review board of Xijing Hospital approved this retrospective study and waived the need for informed consent. This study was conducted in accordance with the Declaration of Helsinki. A computerized CT database was retrospectively searched from January 2013 through June 2016; the key words “type A aortic dissection” and “surgical repair” were used. There were 443 patients in the initial database. ATAAD was defined as any nontraumatic dissection of the aorta proximal to the left subclavian artery presenting within 14 days of symptom onset^22^. A total of 188 patients were excluded for the following reasons: non-acute type A aortic dissection (n = 6); a history of previous aortic operation (n = 32); unavailable preoperative aortic CTA data from other medical institutions (n = 114); CT image quality that could not be evaluated (n = 11); a time interval between admission and emergency surgery repair of more than 24 hours (n = 5); and postoperative mortality (n = 20). In total, 255 patients with ATAAD were finally eligible for the present study. The standard thoracic aortic operations were performed primarily by 2 dedicated principal surgeons, and the procedures were standardized.

### Image Acquisition

A second-generation dual source CT (Somatom Definition Flash, Siemens Healthcare, Forchheim, Germany) with high-pitch spiral scan mode was used for scanning. Patients underwent combined CTA imaging of the neck and aorta in the cranio-caudal direction. The scanning parameters were as follows: tube voltage of 100 kV, pitch of 3.0, slice collimation of 2 × 128 × 0.6 mm by means of a z-flying focal spot, and reference tube current of 300 mAs per rotation. For all scans, patients were in a supine position with both arms up. Each patient received an injection of 70 ml of iopromide (Ultravist 370, 370 mg I/mL, Bayer Schering Pharma, Berlin, Germany) at a flow rate of 5 ml/s, followed by 40 ml of saline solution. Bolus tracking was performed in the suprarenal descending aorta with an attenuation threshold of 100 HU. The raw data were transferred to an external workstation (syngo MMWP VE 36 A, Siemens Healthcare, Forchheim, Germany) for further postprocessing.

### Neurological Evaluation

The in-hospital postoperative neurological symptoms and signs of patients were diagnosed after a detailed clinical assessment by neurologists and further neuroimaging. Patients were considered to have PND if they exhibited onset of focal (stroke) or global (coma) deficits or were found to have a focal infarction confirmed by CT scanning or magnetic resonance imaging of the brain^5–7^. TND was defined as a symptom complex involving postoperative confusion, seizures, agitation, or transient delirium with no structural abnormalities in the brain that were detectable via the usual imaging methods, and resolution of the symptoms generally occurred before hospital discharge^5–7^. The patients were assigned to the TND, PND, or NND group.

### CTA Findings, Definition and Parameters

CCA, ICA and VA were included the analysis, which are directly correlated with the cerebral artery, which supplies cerebral blood, and indirectly correlated with the thoracic aorta. The diameter of the involved aAO was defines as the maximum diameter of the aAO in the section orthogonal to the centreline. A retrograde aAO dissection is a primary intimal tear in the aortic arch or dAO in which the intimal flap extends to the aAO in a retrograde manner without re-entry. The diameter of the entry point was recorded as the maximum diameter in axial or oblique sagittal images. Low enhancement in the unilateral carotid artery was defined as a CT value for the unilateral artery in a slice of the C1 vertebra that was lower than that for the offside artery for more than 100 Hu.

### Detailed CTA Characteristics of CCA Dissection

Due to the complexity of the impaired status, the location, extent and detailed characteristics of the CCA dissection were further analysed. The detailed characteristics of CCA dissection include (1) re-entry and lower enhancement in the false lumen; (2) re-entry and the same enhancement between the true and false lumen; (3) no re-entry but a severely narrowed true lumen; and (4) no re-entry or a severely narrowed true lumen. A severely narrowed (> 75%) true lumen was defined as a minimum diameter of the true lumen that was less than 25% of the CCA diameter in the same section^20^.

### Statistical analyses

Statistical analyses were performed using SPSS software (version 17.0; SPSS, Inc, Chicago, IL, USA). Categorical variables were presented as the number and percentage, and continuous variables were described as the mean ± standard deviation. Statistical significance was determined with the χ^2^ test for categorical variables and one-way analysis of variance for continuous variables. A *P* value < 0.05 was considered significant. Cohen’s Kappa statistics were calculated for interreader agreement, for the assessment of image quality. Univariate analysis was applied to discriminate CTA risk factors for postoperative PND and TND. Significant CTA risk factors were further analysed in a multivariate logistic regression model to identify isolated CTA risk factors for TND and PND. All *P* values and 95% confidence intervals (CIs) were two-tailed.
